# Biohybrid Nanostructured Iron Oxide Nanoparticles and *Satureja hortensis* to Prevent Fungal Biofilm Development

**DOI:** 10.3390/ijms140918110

**Published:** 2013-09-04

**Authors:** Ion Anghel, Alexandru Mihai Grumezescu, Alina Maria Holban, Anton Ficai, Alina Georgiana Anghel, Mariana Carmen Chifiriuc

**Affiliations:** 1 Otorhinolaryngology, Carol Davila University of Medicine and Pharmacy, Traian Vuia no 6, Bucharest 020956, Romania; E-Mails: ionangheldoc@yahoo.com (I.A.); dr_alina.anghel@yahoo.com (A.G.A.); 2 R & D Department, Doctor Anghel Medical Center, Theodor Sperantia Street, Bucharest 30932, Romania; 3 Department of Science and Engineering of Oxide Materials and Nanomaterials, Faculty of Applied Chemistry and Materials Science, Politehnica University of Bucharest, Bucharest 011061, Romania; E-Mails: grumezescu@yahoo.com (A.M.G.); anton_ficai81@yahoo.com (A.F.); 4 Department of Microbiology and Immunology, Faculty of Biology, University of Bucharest, Bucharest 060101, Romania; E-Mail: carmen_balotescu@yahoo.com

**Keywords:** nano-modified wound dressing, *Satureja hortensis* essential oil, fungal biofilm, *Candida albicans*, magnetite nanoparticles, iron oxide

## Abstract

Cutaneous wounds are often superinfected during the healing process and this leads to prolonged convalescence and discomfort. Usage of suitable wound dressings is very important for an appropriate wound care leading to a correct healing. The aim of this study was to demonstrate the influence of a nano-coated wound dressing (WD) on *Candida albicans* colonization rate and biofilm formation. The modified WD was achieved by submerging the dressing pieces into a nanofluid composed of functionalized magnetite nanoparticles and *Satureja hortensis* (SO) essential oil (EO). Chemical composition of the EO was established by GC-MS. The fabricated nanostructure was characterized by X-ray Diffraction (XRD), Transmission Electron Microscopy (TEM), Differential Thermal Analysis (DTA) and Fourier Transform-Infrared Spectroscopy (FT-IR). The analysis of the colonized surfaces using (Scanning Electron Microscopy) SEM revealed that *C. albicans* adherence and subsequent biofilm development are strongly inhibited on the surface of wound dressing fibers coated with the obtained nanofluid, comparing with regular uncoated materials. The results were also confirmed by the assay of the viable fungal cells embedded in the biofilm. Our data demonstrate that the obtained phytonanocoating improve the resistance of wound dressing surface to *C. albicans* colonization, which is often an etiological cause of local infections, impairing the appropriate wound healing.

## 1. Introduction

The use of modified nanostructured surfaces for the design of film-coated surfaces of solid and fiber-based materials provide a new approach to prevent or disrupt the formation of microbial biofilms [1]. *Candida albicans* associated wound infections are frequently associated with burns (28%) [2], and less frequently with non-surgical epithelial injuries (0.8%) [3]. A significant increase was seen in infections attributable to *C. albicans* in surgical site postoperative infections [4]. One of the major complications in *C. albicans* wound infections is biofilm formation, since microorganisms embedded in biofilms are hundred times more resistant to antifungal compounds [5,6], the infection being therefore difficult to eradicate [7,8].

Iron oxide based nanosized materials are of great interest for the biomedical field due to their excellent properties [9], derived from their intrinsic magnetic nature, as well as from the enhanced physico-chemical properties, such as ultra small and controllable size, large surface area to mass ratio, high reactivity, and functionalizable structure [10]. Magnetite (Fe_3_O_4_) has been widely studied for biomedical applications in biological separations [11], drug delivery and targeting [12–15], magnetic resonance imaging [16], hyperthermia [17], cancer treatment [18,19], stabilization of essential oils [20], inhibition of microbial colonization [21] and ferrofluids [9,22]. The magnetic nanoparticles as delivery nanosystems are considered effective new tools to tackle the current challenges in treating infectious diseases, by improving the therapeutic index of antimicrobial drugs, and diminishing the local and systemic side effects including cutaneous irritation, peeling, scaling and gut flora reduction [23].

The essential oils are an interesting alternative for the antimicrobial therapy, acting by multiple mechanisms, including cell wall damages, inhibiting the cell wall or protein synthesis, or interfering with intermediary metabolisms or DNA/RNA synthesis/function [24,25]. However, the therapeutic effects of the essential oils can be impaired by their high volatility, highlighting the necessity of vectoring stabilizing systems. *Satureja hortensis* (SH) is an annual, herbaceous plant belonging to the family Labiatae [26]. The main components of the essential oils of this plant are the carvacrol, thymol, p-cymene, β-caryophyllene, linalool and other terpenoids [27]. The essential oils isolated from various species of Satureja have biological properties such as antimicrobial, antiviral, antispasmodic and antidiarrhoeal [28].

Routinely used wound dressings are essential in any wound care. An ideal wound dressing should be completely biocompatible and skin-friendly, but also unpermissive for microbial development on the wound lesion. We have previously reported the obtaining of modified textile wound dressings coated with functionalized magnetite nanoparticles, with improved antimicrobial and antibiofilm properties, towards both bacterial and fungal strains. Also the functionalized magnetite nanoparticles proved to act as an efficient delivery system for essential oils and some of their major components.

In this study we report the fabrication, characterization and bioevaluation of a novel wound dressing coating, containing iron oxide nanoparticles and *Satureja hortensis* essential oil. These modified wound dressings exhibited improved antimicrobial properties, preventing fungal colonization and biofilm development.

## 2. Results and Discussion

Among the promising approaches to combat biofilm infections is the generation of surface modification of devices to reduce microbial attachment and biofilm development, as well as incorporation of antimicrobial agents to prevent colonization. The essential oils represent a promising alternative to antimicrobial substances, due to their multiple advantages, such as: an easy way of obtaining low mammalian toxicity, quick biodegradability and low probability for the development of bacterial resistance [29]. Recent studies have shown that nanoparticles can be used for the stabilization and prolonged delivery of essential oils and for the enhancement of their activity at the site of infection, thus surpassing some of the main drawbacks for conventional antimicrobial agents, which are the development of multiple drug resistance and adverse side effects.

Our previous studies have demonstrated that *Rosmarinus officinalis* essential oil-coated magnetic nanoparticles strongly inhibited the adherence ability and biofilm development of *C. albicans* and *C. tropicalis* clinical strains [30] on the catheter surface, and usnic acid-coated magnetic nanoparticles strongly inhibited the adherence ability and biofilm development of Staphylococcus aureus on the coverslips surface, opening new perspectives for the design of antimicrobial and antibiofilm surfaces, based on hybrid functionalized nanostructured biomaterials [31]. In this paper we have investigated the antifungal biofilm properties of a modified wound dressing with hybrid nano-coating based on magnetic nanoparticles and SH essential oil.

The SH essential oil isolated by microwave assisted distillation from the aerial parts of *S. hortensis*, was found to be a yellow liquid and the main components were presented in Table 1.

Eleven components were identified in the essential oil of SH. The main constituents of the essential oil of SH are the carvacrol (46.9%), γ-terpinene (38.7%), p-cymene (4.8%), α-terpinene (3.6%) and myrcene (1.3%). Variation in essential oil content and composition of SH essential oils from different origins has been reported in the literature [32]. The reported results support previous literature data [33].

**Figure 1:**
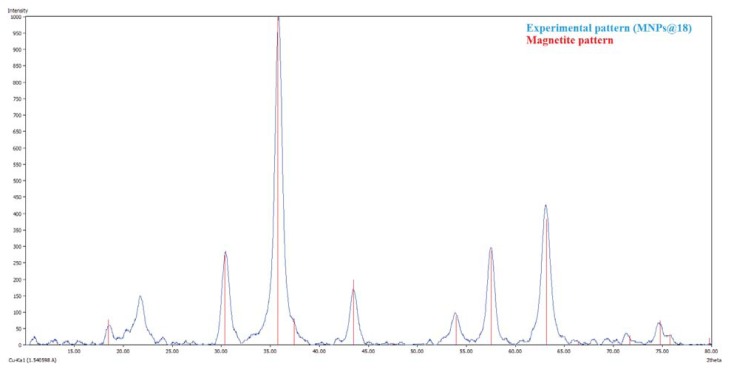
XRD pattern of MNP@18.

XRD pattern (Figure 1) show that the MNP@18 are well-crystalline and exhibit diffraction peaks corresponding to (111), (220), (311), (400), (511) and (440) planes of cubic crystal system. The position and relative intensity of diffraction peaks are same with the standard data for bulk magnetite (JCPDS file No. 19-0629) which further indicates the purity of synthesized MNP@18. XRD supports the data previously reported [34].

**Figure 2:**
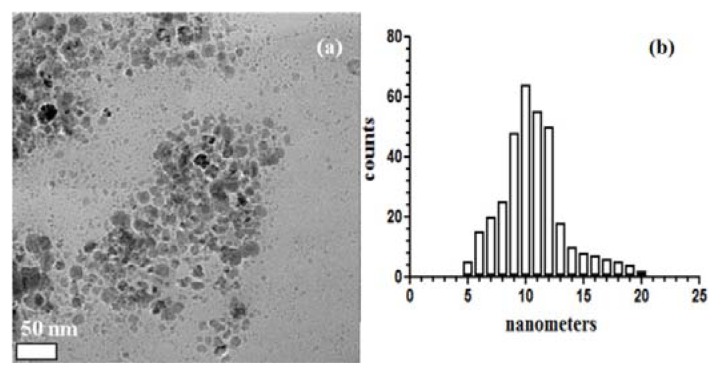
Transmission electron microscopy (TEM) images of MNPs@18 (**a**) and histogram showing the size distribution of the MNPs@18 (**b**).

The TEM images of the MNP@18 were used to determine the shape, size and uniformity of the particles. Figure 2a,b that the particles are spherical and polydispersed with an average size of 10 nm.

**Figure 3:**
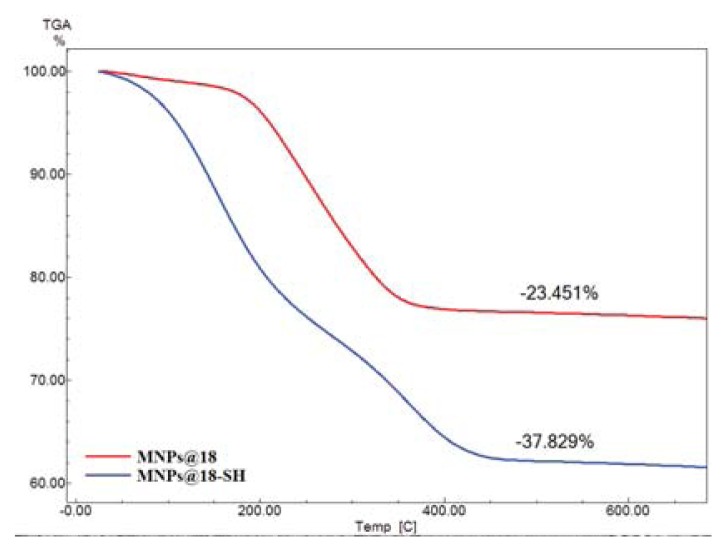
Thermogravimetric (TG) analysis of MNP@18-SH and MNP@18.

The TGA thermograms revealed continuous weight loss for C18 and SH (Figure 3). The weight losses are 23.45% for MNP@18, and 37.82% for of MNP@18-SH. The results confirmed the attachment and stabilization of the volatility of the SH essential oil on MNP@18 surface. The SH essential oil content was estimated as the difference between the weight loss for the region at approximately 500 °C for MNP@18-SH and MNP@18, and it was approximately 14.37%.

**Figure 4:**
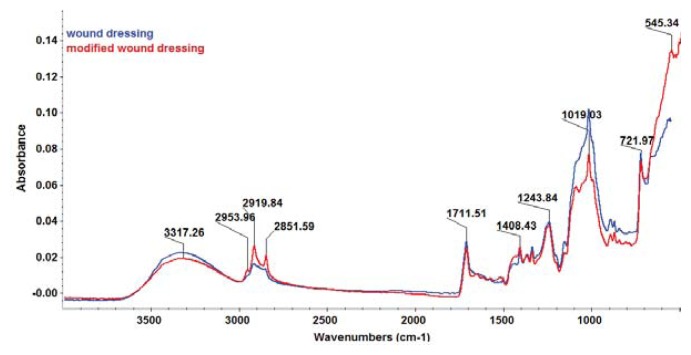
Fourier Transform-Infrared Spectroscopy (FT-IR) spectra of (modified) wound dressing (WD).

The FT-IR spectrum of modified wound dressing (Figure 4) showed characteristic band of iron oxide at ~545 cm^−1^ attributed to the stretching vibration of Fe–O bonds [35] and characteristic bands assigned to stretching vibration of C–H from organic coating (C18) at about 2919 and 2851 cm^−1^.

Recent studies have proved that the major compounds identified in the composition of SH essential oil exhibit antimicrobial properties [36,37]. The essential oils extracted from different aerial parts of SH (*i.e.*, budding, full flowering, immature fruit, and ripened fruit stages) exhibited strong antibacterial activities against a wide range of bacterial and fungal strains (including *S. aureus*, *E. coli* and *C. albicans*), clearly demonstrating their potential to be used in the management of microbial infections [38–41]. Other studies have shown that the amount of EOs extracted from different air dried samples were quite similar varying from 1.8% in case of ripened fruit stage to 2.5% at full flowering, as well as the chemical composition, which was quite consistent, the δ-terpinene being the major compound of the EO at all developmental stages, except the ripened fruit stage when it was replaced by carvacrol [37]. These two compounds have been also found in major percentages in the composition of the EO used in the present study.

The SH essential oils could also contribute to the development of environmentally safer alternatives to protect the spoilage of food products from pathogenic and saprophytic fungi, by inhibiting the mycelial growth of *Alternaria mali* Roberts and *Botrytis cinerea* Pers fungi, and also exhibiting a fungicidal effect against these phytopathogenic species [42].

In our study, viable cell counts results revealed that MNP@18-SH coated WD exhibited significant antimicrobial properties, disrupting fungal adherence and biofilm formation.

**Figure 5:**
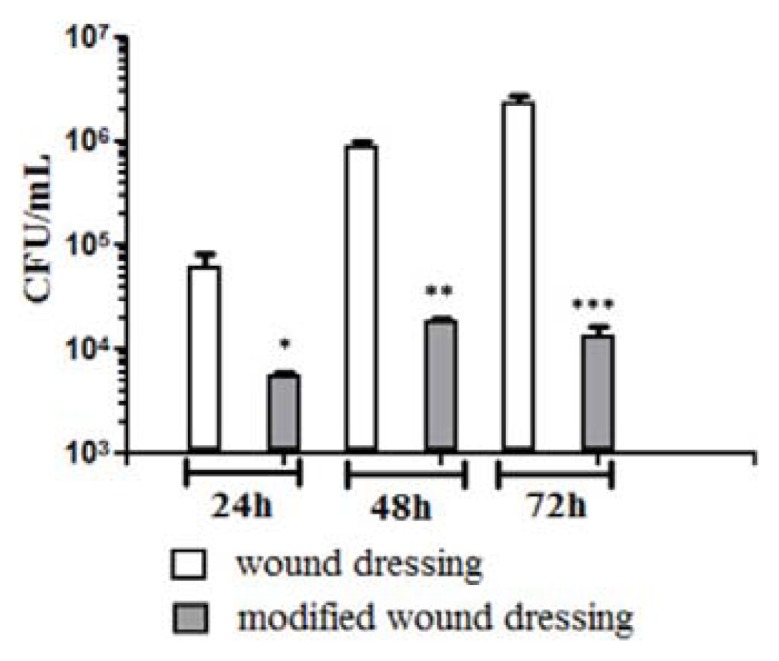
Graphic representation of viable cell counts analysis after removing *C. albicans* biofilm embedded cells at 24 h, 48 h and 72 h post inoculation of control and nanobiocoated WDs. * *p* < 0.05, ** *p* < 0.01, *** *p* < 0.001 samples *vs.* WD control.

**Figure 6:**
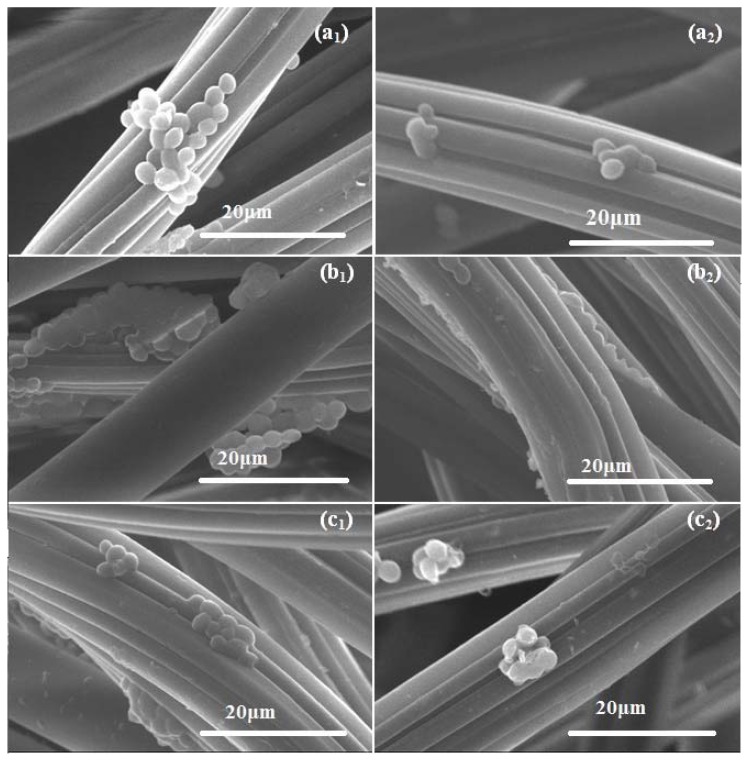
SEM micrographs indicating the *C. albicans* biofilm development comparatively on control WDs (after 24 h—**a_1_**, 48 h—**b_1_** and 72 h—**c_1_** incubation time) and on MNP@18-SH coated WDs (after 24 h—**a_2_**, 48 h—**b_2_**. and 72 h—**c_2_** incubation) (2500×). The *Candida* biofilms developed on the coated WDs are strongly damaged and drastically reduced.

On the regular WD fibers, the kinetics of *C. albicans* biofilm registered an ascending trend from 24 h to 72 h, as revealed by the increasing number of biofilm embedded viable cells. The biofilm formation on the nanomodified WDs was impaired in its early as well as mature phases, quantified at 24 h, 48 h and 72 h (Figure 5), therefore the nano-coating stabilizes and preserves the antimicrobial activity of the essential oil. Viable cell counts data were also confirmed by the microscopic examination of the biofilm architecture and development. The scanning electron microscopy images showed that *C. albicans* formed yeast microcolonies embedded in an extracellular mathrix on the surface of control WDs, this ability being abolished when using nanobiocoated WD surfaces colonized for 24 h, 48 h and 72 h (Figure 6). The antimicrobial activities of SH essential oil could be explained by the high content in phenolic compounds, such as thymol and carvacrol or p-cymene, whose antimicrobial effect is due to damages induced in the membrane integrity, causing changes in pH homeostasis and also in the equilibrium of inorganic ions [39]. Although p-cymene is not reported in the literature as having antimicrobial activity, it increases the antimicrobial activity of thymol or carvacrol [43], by destabilizing the cytoplasmic membrane of microbial cell [44]. This synergic activity of the active compounds has motivated us to use the essential oil whole extract instead of individual compounds. The antibiofilm activity of the modified wound dressing was preserved on the entire duration of the experiment, proving that the nanoparticles acted as an efficient stabilization and long lasting release vehicle for the essential oil.

## 3. Experimental Section

### 3.1. Materials

All chemicals were used as received. FeCl_3_, FeSO_4_·7H_2_O, NH_4_OH (25%), and CH_3_OH were purchased from Sigma-Aldrich ChemieGmbh (Munich, Germany). General-use 10 × 10 mm rayon/polyester based wound dressings were provided from Doctor Anghel’s Medical Center.

### 3.2. Synthesis of Functionalized Magnetite Nanostructure

Magnetite nanostructure was prepared by wet chemical precipitation from aqueous iron salt solutions by means of alkaline media [13,45,46]. Synthesis of functionalized magnetite nanostructure involves several steps. Briefly, magnetic nanoparticles of approximately 10 nm diameter were precipitated in alkaline solution of sodium stearate (C_18_) from solution of Fe(II) and Fe(III) according to our recently published paper [47]. After the precipitation of functionalized magnetite nanocrystals (MNP@18), it was repeatedly washed with methanol and separated with a strong NdFeB permanent magnet.

### 3.3. Extraction and Analysis of *Satureja hortensis (SH)* Essential Oil

The essential oil microwave assisted extraction was performed in a Neo-Clevenger type apparatus and its chemical composition was settled by GC–MS analysis. Gas chromatographic analysis was performed using an Agilent 6890 Series GC System (Agilent Technologies Inc., Santa Clara, CA, USA) gas chromatograph fitted with a splitless injector for a low background under a column head pressure of 12.5 psi and H_2_ as carrier gas at a flow rate of 1.2 mL/min. Oven temperature was programmed from 50 °C to 300 °C at 5 °C/min. Injector and detector temperatures were 250 °C. A capillary column DB5-MS fused-silica J&W Scientific Inc. (Krackeler Scientific, Inc., Albany, NY, USA) was used (30 m × 0.25 mm i.d.; 0.25 μm film). Detection was carried out with a 5973 mass-selective single quadrupole detector (Agilent technologies Inc., Santa Clara, CA, USA). Operation control and the data process were carried out by Agilent Technologies ChemStation software (Santa Clara, CA, USA). The mass spectrometer was calibrated before use with perfluorotributylamine (PFTBA) as a calibration standard.

### 3.4. Fabrication of Functionalized Magnetite Biohybrid Nanostructure

MNP@18 (100 mg) and 100 μL of SH were solubilized in 2 mL of chloroform and mixed until complete evaporation of chloroform was reached. According to our previous published work [20], we observed that this ratio (100 mg of MNP@18 and 100 μL of essential oil) is the most appropriate for the efficient stabilization of the most essential oils.

This step was repeated three times for the uniform loading of HS in the MNP@18. After 72 h the prepared MNP@18-SH was analyzed by TGA to estimate the amount of SH essential oil entrapped into the MNP@18 [30,31].

### 3.5. Fabrication of Modified Wound Dressing

After 72 h of drying at room temperature, the layer of MNP@18-SH on the wound dressing material was achieved by submerging the dressing pieces (10 × 10 mm) in 5 mL of MNP@18-SH fluid (MNP@18-SH:CHCl_3_ = 1 mg/mL), and then the dressing pieces have been extemporaneously dried at room temperature. The rapid drying was facilitated by the convenient volatility of chloroform. The modified wound dressing specimens were sterilized by ultraviolet irradiation for 20 min.

### 3.6. Characterization

#### 3.6.1. TEM

The transmission electron microscopy (TEM) images were obtained on finely powdered samples using a Tecnai™ G2 F30 S-TWIN high resolution transmission electron microscope from FEI Company (Hillsboro, OR, USA). The microscope was operated in transmission mode at 300 kV with TEM point resolution of 2 Å and line resolution of 1 Å. The fine powder was dispersed into pure ethanol and ultrasonicated for 15 min. After that, diluted sample was put onto a holey carbon-coated copper grid and left to dry before TEM analysis.

#### 3.6.2. XRD

X-ray diffraction analysis was performed on a Shimadzu XRD 6000 diffractometer at room temperature. In all the cases, Cu Kα radiation (λ = 15,406 Å at 15 mA and 30 kV) was used. The samples were scanned in the Bragg angle 2θ range of 10–80 degree.

#### 3.6.3. FT-IR

A Nicolet 6700 FT-IR spectrometer (Thermo Nicolet, Madison, WI, USA) connected to the software of the OMNIC operating system (Version 8.2; Thermo Nicolet, Madison, WI, USA) was used to obtain FT-IR spectra of the modified wound dressings. The samples were placed in contact with attenuated total reflectance (ATR) on a multibounce plate of ZnSe crystal at controlled ambient temperature (25 °C). FT-IR spectra were collected in the frequency range of 4000–650 cm^−1^ by co-adding 32 scans and at a resolution of 4 cm^−1^ with strong apodization. All spectra were ratioed against a background of an air spectrum.

#### 3.6.4. TG analysis

The thermogravimetric (TG) analysis of the MNP@18 and MNP@18-SH was followed with a Netzsch TG 449C STA Jupiter instrument (Netzsch, Selb, Germany). Samples were screened with 200 mesh prior to analysis, placed in an alumina crucible, and heated at 10 K min^−1^ from room temperature to 800 °C, under the flow of 20 mL min^−1^ of dried synthetic air (80% N_2_ and 20% O_2_).

#### 3.6.5. SEM

SEM analysis was performed on a HITACHI S2600N electron microscope, at 20 keV, in secondary electrons fascicle, on samples covered with a thin silver layer. After 24 h, 48 h and 72 h incubation period, WDs were washed gently with sterile PBS for not disturbing the biofilm, and fixed by immersing each sample in methanol for 5 s. After fixation, samples were allowed to air dry and examined by SEM. Each experiment was performed in triplicate and repeated on at least three separate occasions. For each sample at least three microscopic fields were randomly analyzed by two independent observers. Micrographs considered significant for both observers were selected.

### 3.7. Strains and Culture Conditions

*C. albicans* ATCC 10231 was purchased from ATCC (American Type Culture Collection, Manassas, VA, USA) and cultured using Sabouraud Agar and Sabouraud broth (Acumedia, Bucharest, Romania). Fungal inoculum was grown overnight in Sabouraud broth and diluted ~1000 times in the same medium, for reaching a density of 10^2^–10^3^ CFU/mL.

### 3.8. *In Vitro* Fungal Biofilm Development

Biofilm formation was assessed in 6 multi-well plates (Nunc, St. Louis, MO, USA), using a static model for monospecific biofilms development. Control WD and MNP@18-SH coated WD pieces of 1 cm × 1 cm were sterilized by exposure to direct UV light for 20 min and distributed in 6 multi-well plates (one per well). The *C. albicans* inoculums (2 mL) with standardized density were added in each well, to completely cover the WD pieces. Samples were incubated for 24 h at 37 °C. Biofilms were analyzed by viable cell count assay. Briefly, after 24 h incubation the culture medium was removed and the pieces of WD washed with sterile PBS (phosphate buffered saline), in order to remove unattached bacteria. WD samples were placed in fresh medium and incubated for other additional 24 h, 48 h and 72 h. After the incubation period wound dressing pieces were gently washed with sterile PBS for not disturbing the biofilm and placed in 1.5 mL Eppendorf tubes containing 750 μL PBS. Samples were vigorously mixed by vortexing for 30 s and sonicated for 10 s in order to disperse biofilm cells into the suspension. Serial ten-fold dilutions were achieved and plated on Sabouraud Agar for viable cell counts assay. Experiments were performed in triplicate and repeated on three separate occasions.

### 3.9. Statistical Analysis

Data were analyzed using GraphPadIn Stat and Prism softwares, by applying One-way Analysis of Variance (ANOVA) test. *p* values lower than 0.05 were considered significant.

## 4. Conclusions

Classical wound dressings were successfully modified by coating with a novel nanobiosystem based on functionalized magnetite nanoparticles and SH essential oil. The essential oil was extracted by microwave assisted Neo-Clevenger apparatus and characterized by GC–MS. TEM, XRD, TGA and FT-IR characterization of the fabricated nanostructured coating demonstrated its nanosized uniform structure. The biological assay revealed that the newly fabricated nanobiocoating exhibited antimicrobial properties, rendering the wound dressings fibers more resistant to fungal cells adherence and biofilm development. Our results proved that the obtained nanobiocoating combining the excellent properties of iron oxide nanoparticles and the essential oil with antimicrobial properties could represent a novel and successful alternative for inhibiting fungal adhesion and biofilm formation on medical devices and other clinically relevant materials and any surfaces.

## Figures and Tables

**Table 1:** GC-MS analysis of *Satureja hortensis* (SH) essential oil.

No.	Compound	Retention Index 1	(%)
1	α-thujene	927	1
2	α-pinene	940	0.9
3	β-pinene	984	0.7
4	myrcene	1000	1.3
5	α-terpinene	1019	3.6
6	p-cymene	1028	4.8
7	γ-terpinene	1057	38.7
8	linalool	1103	0.9
9	carvacrol	1301	46.9
10	β-caryophyllene	1413	0.1
11	β-bisabolone	1504	0.7

1 RI is the retention indices in elution order from DB-5 column.
